# Multidrug-Resistant *Salmonella enterica* Serovar Infantis, Israel

**DOI:** 10.3201/eid1611.100100

**Published:** 2010-11

**Authors:** Ohad Gal-Mor, Lea Valinsky, Miriam Weinberger, Sara Guy, Joseph Jaffe, Yosef Ilan Schorr, Abraham Raisfeld, Vered Agmon, Israel Nissan

**Affiliations:** Author affiliations: Sheba Medical Center, Tel-Hashomer, Israel (O. Gal-Mor);; Ministry of Health Laboratories, Jerusalem, Israel (L. Valinsky, S. Guy, J. Jaffe, Y.I. Schorr, A. Raisfeld, V. Agmon, I. Nissan);; Assaf Harofeh Medical Center, Zerifin, Israel (M. Weinberger)

**Keywords:** Bacteria, Salmonella, enteric infections, foodborne infections, Salmonella enterica, Infantis, serovars, antibicrobial resistance, Israel, dispatch

## Abstract

To determine whether rapid emergence of *Salmonella enterica* serovar Infantis in Israel resulted from an increase in different biotypes or spread of 1 clone, we characterized 87 serovar Infantis isolates on the genotypic and phenotypic levels. The emerging strain comprised 1 genetic clone with a distinct pulsed-field gel electrophoresis profile and a common antimicrobial drug resistance pattern.

Nontyphoid *Salmonella enterica* (NTS) is a common cause of foodborne illnesses worldwide. In industrialized countries, *S. enterica* serovars Enteritidis and Typhimurium are responsible for most NTS infections (*1*). In Israel, the distribution of NTS infections differs from the global epidemiology for NTS by having a larger representation of serogroups C1 and C2 (serovars Virchow, Hadar, and Infantis) in addition to serovars Enteritidis and Typhimurium (*2**,**3*).

Analysis of annual trends of NTS infections in Israel during 1995–2009 shows a steady decrease in the incidence of these infections, from 86.9 cases/100,000 persons in 1995 to 31.4/100,000 in 2005. During this period, the predominant serovars were Enteritidis, Typhimurium, Virchow, and Hadar, followed by Infantis. Since 2006, annual incidence of NTS has started to increase, rising to 44.0 cases/100,000 persons in 2009. This trend coincided with a sharp increase in incidence of serovar. Infantis from 1.2 cases/100,000 persons in 2001 to 14.7/100,000 in 2009, a 12-fold rise (Figure 1, panel A). The proportion of serovar Infantis increased from <10% of NTS in 1995–2005 to 34% in 2009 (Figure 1, panel B). Furthermore, this steep increase in serovar Infantis from clinical (human) sources correlated with an elevated frequency of serovar Infantis from poultry that became apparent after 2006. Serovar Infantis became the predominant serotype in poultry during 2007–2009, while the prevalences of serovars Enteritidis, Typhimurium, Virchow, Bredeney, Newport, and Paratyphi B var. Java decreased (Figure 1, panel C).

**Figure 1:**
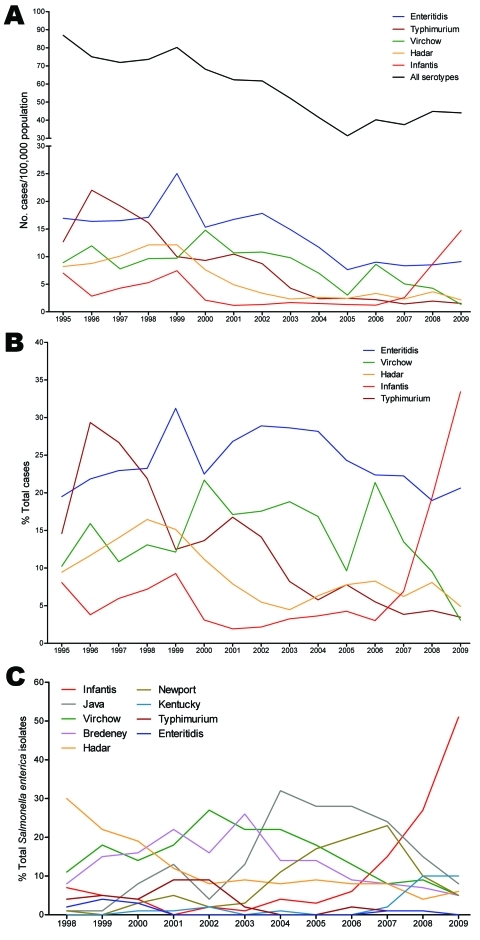
Salmonellosis epidemiology in Israel, 1995–2009. A) Annual incidence of salmonellosis in Israel. Laboratory-confirmed cases of *Salmonella* infections per 100,000 population caused by all *Salmonella* serotypes (black) and by the 5 leading serotypes in Israel. B) The relative contribution (in percentages) of each serotype to the total annual number of *Salmonella* serotypes. *Salmonella* infection incidences were constructed according to the number of human *Salmonella* isolates submitted to the Government Central Laboratories during January 1, 1995–December 31, 2009 (after excluding repeated isolates from the same patient). Data on the Israeli population were derived from the publications of the Israeli Bureau of Statistics. C) Prevalence of *S. enterica* serovar Infantis and other leading serotypes in poultry. The proportion of different *Salmonella* serotypes as percentage from the total *Salmonella* isolates in poultry was analyzed according to routine surveillance in poultry processing plants conducted by veterinary services in 1998–2009. *Salmonella* isolates have been received, identified, and documented in the National *Salmonella* Reference Center of Israel.

## The Study

**Figure 2:**
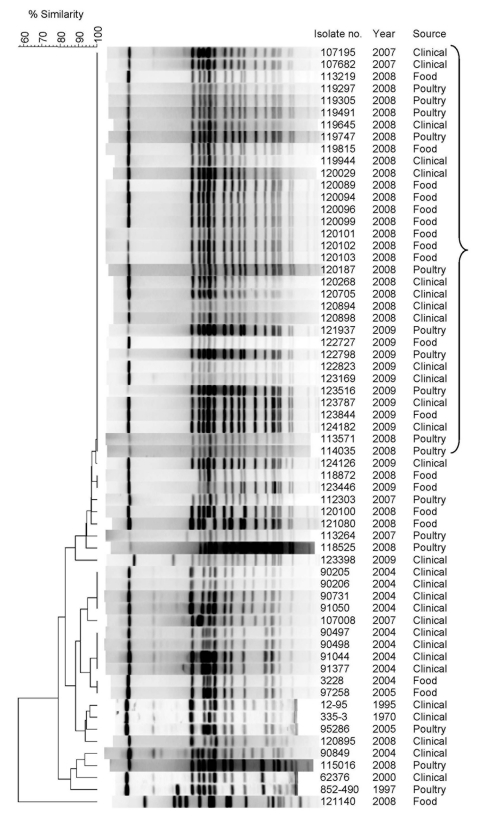
Pulsed-field gel electrophoresis (PFGE) patterns of *Salmonella enterica* serovar Infantis isolates from clinical, food, and poultry sources isolated in Israel, 1970–2009, showing a high degree of clonality. Isolate number, year of isolation, and source are indicated. Bracket indicates I1 pulsotype pattern. Macrodigestion performed using *Xba*I restriction enzyme and genetic similarity (in %) was based on dice coefficients. PFGE was conducted according to the standardized *Salmonella* protocol Centers for Disease Prevention and Control PulseNet as described (*4*) by using *S. enterica* ser. Braenderup H9812 strain as a molecular size standard. Because of space limitations, only 34/58 pulsotype I1 clones are shown. A complete list is provided in Table A1.

Molecular analysis was used to study whether the rapid emergence of *S. enterica* ser. Infantis resulted from a general increase in different biotypes or a successful spread of 1 clone. Seventy-one randomly selected isolates of *S. enterica* ser. Infantis identified in Israel during 2007–2009 (21 human sources, 28 poultry sources, and 22 food sources) and 16 historical strains isolated during 1970–2005 (12 human sources, 2 poultry sources, and 2 food sources) were subjected to pulsed-field gel electrophoresis (PFGE). Macrorestriction with the *Xba*I enzyme discriminated the isolates into 23 distinct profiles (pulsotypes), designated I1–I23. Although the historical isolates showed high diversity in their PFGE patterns, most (58/71, 82%) recent (2007–2009) isolates were homogeneous and showed an indistinguishable PFGE profile (pulsotype I1), which was not found among the historical isolates (Figure 2; Table A1). These results indicate that most of the emerging isolates belong to 1 genetic clone that probably started to spread in Israel sometime during 2005–2007. Furthermore, comparison of the I1 pulsotype with other PFGE profiles through PulseNet (www.cdc.gov/pulsenet/) and PulseNet Europe (www.pulsenetinternational.org/networks/europe.asp) indicated a pattern not reported elsewhere, suggesting the emerging clone is endemic to Israel.

To further characterize the isolates, we performed susceptibility tests to 16 antimicrobial compounds. Overall, resistance to 11 antimicrobial agents was detected (Table; Table A1). Two clear differences were found between the strains isolated before and after 2007. First, although 6/16 (38%) of the historical strains were sensitive to all tested antimicrobial agents and 5/16 (31%) were resistant to only 1 (nitrofurantoin), none of the 2007–2009 isolates were sensitive to all of the tested antimicrobial agents. Most (68/71, 96%) of the recent isolates were resistant to >3 antimicrobial agents, which suggests a process of resistance acquisition over time. Second, whereas isolates from 1970–2005 did not share any obvious resistance pattern, most (66/71, 93%) of the 2007–2009 strains showed a combined resistance pattern to nalidixic acid, nitrofurantoin, and tetracycline with or without additional resistance to trimethoprim/sulfamethoxazole (Table). The convergence of the recent serovar Infantis clones to a dominant resistance pattern is consistent with their common PFGE profile and shows that they share high similarity on phenotypic and genotypic levels.

**Table:** Antimicrobial drug resistance patterns of Salmonella enterica serovar Infantis isolates, sorted by isolation year, Israel*

Antimicrobial drug resistance profile	PFGE pattern												
	1970–2005			2007			2008			2009			Total
	I1	D		I1	D		I1	D		I1	D		
Ampicillin, cefuroxime, ceftriaxone, cephalothin, nitrofurantoin, trimethoprim/sulfamethoxazole		1											1
Ampicillin, cefepime, ceftriaxone, cephalothin, cefuroxime, tobramycin		1											1
Nitrofurantoin		5											5
Nitrofurantoin, tetracycline					1								1
Levofloxacin, nalidixic acid								1					1
Nalidixic acid, nitrofurantoin, tetracycline				1	1		37	3		8	3		53
Nalidixic acid, nitrofurantoin, tetracycline, cephalothin								1					1
Nalidixic acid, nitrofurantoin, tetracycline, trimethoprim/sulfamethoxazole		2		1	1		10			1			15
Nalidixic acid, tetracycline, trimethoprim/sulfamethoxazole								1					1
Nalidixic acid, tetracycline								1					1
Nalidixic acid, trimethoprim/sulfamethoxazole		1											1
Sensitive to all tested antimicrobial drugs		6											6
Total		16		2	3		47	7		9	3		87

*PFGE, pulsed-field gel electrophoresis; I1, emerging PFGE pattern; D, different from the emerging pattern.

Next, we characterized the molecular mechanisms responsible for the common antimicrobial drug–resistance phenotype. In bacteria, an efficient means of acquisition and dissemination of resistance genes is through mobile genetic elements such as plasmids, transposons, or integrons (*5*). Plasmid analysis for 15 emerging (2007–2009) and 7 historical (1970–2005) randomly selected isolates demonstrated that all possessed 1 large plasmid of ≈100 kb. To identify antimicrobial drug resistance genes that are possibly encoded on this plasmid, mating experiments were conducted with a plasmid-free, rifampin-resistant *Escherichia coli* J5–3 strain and recent *S. enterica* ser. Infantis isolates harboring tetracycline, nalidixic acid, and nitrofurantoin resistance genes. Conjugation experiments showed the obtained *E. coli* transconjugants received the large (≈100-kb) plasmid and acquired the tetracycline resistance phenotype but remained susceptible to nalidixic acid and nitrofurantoin. We concluded the tetracycline resistance gene(s) is encoded on the conjugative plasmid. Molecular analysis by PCR showed the *tetA* gene encoded within the Tn*1721* transposon in 6 of 6 randomly selected emerging isolates but in only 1 of 5 older historical strains.

We examined class 1 integrons using PCR primers designed to amplify the variable region of class 1 integrons. All 6 recent isolates bore 1 integron with a variable region of ≈1 kb. Sequencing of the resulting amplicon showed the *dfrA1* gene cassette conferring resistance to trimethoprim–sulfamethoxazole followed by the *orfC* gene of unknown function. In contrast, 3/5 historical isolates did not possess any integron, and 2/5 contained a disparate integron with a variable region of ≈1.3 kb. Sequencing analysis indicated a different cassette encoded by the aminoglycoside adenyltransferase *aadA1* gene conferring resistance to spectinomycin and streptomycin.

Resistance to quinolones is often associated with point mutations in the quinolone-resistance determining region of the *gyrA* gene (*6*). To examine this possibility, we determined the *gyrA* sequence from 6 recent naladixic acid–resistant and 4 naladixic acid–sensitive isolates. All resistant clones showed the same nucleotide substitution from guanine to thymine at position 259 (G259T) in the *gyrA* gene, resulting in the exchange of asparagine in position 87 to tyrosine (Asp87Tyr) in the quinolone resistance–determining region domain. No mutations were found in the *gyrA* sequence of the naladixic acid–sensitive isolates, suggesting that the Asp87Tyr point mutation is responsible for the observed naladixic acid–resistance phenotype.

## Conclusions

It is likely that environmental selective pressure caused by use of antimicrobial drugs has led to the distribution of appropriate resistant genes. Nitrofurans and sulfonamides, for example, have been widely used to treat infections and promote growth of livestock (*7*). Because the emerging clone was dominant in all levels of the food chain, including broiler chickens, it is possible that the emerging clone was originally introduced from a poultry source. Recent studies from other countries identified healthy poultry as a potential reservoir of *S. enterica* ser. Infantis (*8**–**10*).

Molecular and phenotypic characterization of recent *S. enterica* ser. Infantis isolates from different sources and regions in Israel showed high homogeneity of emerging isolates that differ genetically and phenotypically from previously isolated strains. We showed that the emerging clone is multidrug resistant and is characterized by a large conjugative plasmid harboring the Tn*1721* transposone and *tetA* gene, which provides reduced susceptibility to tetracyclines. Additional characteristics include a class 1 integron containing the *dfrA1* cassette, a *gyrA* mutation that mediates nalidixic acid resistance and furthers resistance to nitrofurantoin. Our results suggest the recent emergence of serovar Infantis is an outcome of a clonal expansion and establishment of a specific biotype that took place during a relatively short period. Virulence mechanisms contributing to this phenomenon are the subject of an ongoing study.

## Appendix

**Table A1:** List of the examined *Salmonella enterica* serovar Infantis isolates, showing source, isolation date, PFGE pattern, and antimicrobial drug susceptibility test results, Israel*

Strain no.	Source	Isolation date	PFGE pattern	Susceptibility
335–3	Archive†	1970	I19	S
12–95	Archive†	1995	I20	S
852–490	Poultry	1997 Sep 19	I23	nal,sxt
62376	Clinical (stool)	2000 Oct 24	I22	nal, fd, tet, sxt
90731	Clinical (stool)	2004 Apr 25	I4	S
91050	Clinical (stool)	2004 Apr 25	I4	S
90205	Clinical (stool)	2004 May 5	I4	S
90206	Clinical (stool)	2004 May 12	I4	S
90497	Clinical (stool)	2004 Jun 3	I2	amp, rox, ctr, cf, fd, sxt
90498	Clinical (stool)	2004 Jun 3	I2	amp, fep, ctr, cf, fd, rox, nn
3228	Food	2004 Jun 13	I3	fd
90849	Clinical (stool)	2004 Jun 21	I7	nal, fd, tet, sxt
91044	Clinical (stool)	2004 Jul 19	I2	fd
91377	Clinical (stool)	2004 Aug 4	I2	fd
95286	Poultry	2005 Mar 28	I21	fd
97258	Food	2005 Aug 22	I3	fd
107008	Clinical (stool)	2007 Jan 8	I5	fd, tet
107195	Clinical (stool)	2007 Jan 15	I1	nal, fd, tet, sxt
107682	Clinical (stool)	2007 Feb 15	I1	nal, fd, tet
112303	Poultry	2007 Nov 8	I9	nal, fd, tet
113264	Poultry	2007 Dec 12	I12	nal, fd, tet, sxt
113571	Poultry	2008 Jan 7	I1	nal, fd, tet
114035	Poultry	2008 Jan 29	I1	nal, fd, tet
114256	Poultry	2008 Feb 17	I1	nal, fd, tet
114509	Poultry	2008 Mar 9	I1	nal, fd, tet
115016	Poultry	2008 Apr 13	I8	nal,tet
115593	Poultry	2008 May 18	I1	nal, fd, tet
115991	Poultry	2008 Jun 1	I1	nal, fd, tet
121162	Food	2008 Sep 8	I1	nal, fd, tet
118525	Poultry	2008 Sep 10	I14	nal, fd, tet
118872	Poultry	2008 Oct 5	I13	nal, fd, tet, cf
121056	Food	2008 Oct 7	I1	nal, fd, tet, sxt
121061	Food	2008 Oct 12	I1	nal, fd, tet
119297	Poultry	2008 Oct 19	I1	nal, fd, tet
119305	Poultry	2008 Oct 26	I1	nal, fd, tet
121078	Food	2008 Oct 30	I1	nal, fd, tet, sxt
121080	Food	2008 Oct 30	I10	nal, fd, tet
119491	Poultry	2008 Nov 2	I1	nal, fd, tet
119645	Clinical (stool)	2008 Nov 4	I1	nal, fd, tet
121093	Food	2008 Nov 4	I1	nal, fd, tet
119747	Poultry	2008 Nov 6	I1	nal, fd, tet
119944	Clinical (stool)	2008 Nov 10	I1	nal, fd, tet
121102	Food	2008 Nov 10	I1	nal, fd, tet
120029	Clinical (stool)	2008 Nov 12	I1	nal, fd, tet
119815	Poultry	2008 Nov 16	I1	nal, fd, tet
121116	Food	2008 Nov 17	I1	nal, fd, tet
120309	Clinical (stool)	2008 Nov 20	I1	nal, fd, tet
120187	Poultry	2008 Nov 21	I1	nal, fd, tet, sxt
120189	Poultry	2008 Nov 21	I1	nal, fd, tet
120173	Clinical (stool)	2008 Nov 24	I1	nal, fd, tet
120191	Poultry	2008 Nov 24	I1	nal, fd, tet, sxt
120186	Clinical (stool)	2008 Nov 25	I1	nal, fd, tet
120195	Poultry	2008 Nov 27	I1	nal, fd, tet
120089	Food	2008 Nov 30	I1	nal, fd, tet
120091	Food	2008 Nov 30	I1	nal, fd, tet, sxt
120094	Food	2008 Nov 30	I1	nal, fd, tet, sxt
120096	Food	2008 Nov 30	I1	nal, fd, tet
120099	Food	2008 Nov 30	I1	nal, fd, tet
120100	Food	2008 Nov 30	I11	lvx, nal
120101	Food	2008 Nov 30	I1	nal, fd, tet
120102	Food	2008 Nov 30	I1	nal, fd, tet, sxt
120103	Food	2008 Nov 30	I1	nal, fd, tet
120268	Clinical (stool)	2008 Nov 30	I1	nal, fd, tet
120314	Poultry	2008 Nov 30	I1	nal, fd, tet
120321	Poultry	2008 Nov 30	I1	nal, fd, tet
121135	Food	2008 Dec 1	I1	nal, fd, tet, sxt
120229	Poultry	2008 Dec 3	I1	nal, fd, tet, sxt
120233	Poultry	2008 Dec 3	I1	nal, fd, tet
120540	Clinical (stool)	2008 Dec 3	I1	nal, fd, tet
121140	Food	2008 Dec 3	I16	nal, tet, sxt
120705	Clinical (stool)	2008 Dec 10	I1	nal, fd, tet
120895	Clinical (stool)	2008 Dec 17	I6	nal, fd, tet
120894	Clinical (stool)	2008 Dec 18	I1	nal, fd, tet
120898	Clinical (stool)	2008 Dec 18	I1	nal, fd, tet, sxt
113219	Poultry	2008 Dec 27	I1	nal, fd, tet
123446	Food	2009 Jan 13	I13	nal, fd, tet
121937	Poultry	2009 Jan 28	I1	nal, fd, tet
122727	Food	2009 Mar 9	I1	nal, fd, tet
123169	Clinical (abscess)	2009 Apr 3	I1	nal, fd, tet
122798	Poultry	2009 Apr 5	I1	nal, fd, tet
122823	Clinical (stool)	2009 Apr 7	I1	nal, fd, tet
123398	Clinical (stool)	2009 May 3	I18	nal, fd, tet
123516	Poultry	2009 May 17	I1	nal, fd, tet
124182	Clinical (blood)	2009 May 26	I1	nal, fd, tet
123787	Clinical (wound)	2009 Jun 5	I1	nal, fd, tet, sxt
123844	Food	2009 Jun 12	I1	nal, fd, tet
124126	Clinical (stool)	2009 Jun 17	I17	nal, fd, tet

*Eighty-seven randomly selected *S. enterica* ser. Infantis isolates from human, food, and poultry sources were selected for PFGE analysis and antimicrobial drug susceptibility test. Because *Salmonella* spp. are reportable pathogens in Israel, the strains were submitted to the Government Central Laboratories by various microbiology laboratories throughout Israel. The National *Salmonella* Reference Center did final serologic identification. Isolate number, their source, isolation date, and PFGE pattern are indicated. Antibacterial drug susceptibility was tested by using the VITEK 1 system (bioMérieux S.A., Marcy l'Etoile, France) and the GNS-210 VITEK card for gram-negative susceptibility testing. *Escherichia coli* ATTC 25922 was used as a reference strain. The tested antibacterial agents were amoxicillin/clavulanic acid; ampicillin (MIC >32 μg/mL); rox, cefuroxime; cefepime (MIC >32 μg/mL); ceftriaxone (MIC >64 μg/mL); cefuroxime (MIC ≥32 μg/mL); cephalothin (MIC >32 μg/mL); ciprofloxacin; gentamicin; imipenem; levofloxacin (MIC >8 μg/mL); nalidixic acid (MIC >32 μg/mL); nitrofurantoin (MIC >128 μg/mL); piperacillin/tazobactam; tetracycline (MIC >16 μg/mL); tobramycin (MIC >16 μg/mL); and trimethoprim/sulfamethoxazole (MIC range 80–320 μg/mL). PFGE, pulsed-field gel electrophoresis; S, sensitive to all examined antibacterial drugs; nal, nalidixic acid; sxt, trimethoprim/sulfamthoxazole; fd, nitrofurantoin; tet, tetracycline; amp, ampicillin; fep, cefepime; ctr, ceftriaxone; cf, cephalothin; nn, tobramycin; lvx, levofloxacin. †National *Salmonella* spp. archive, source unknown.
